# Supplementary material for: Matchtigs: minimum plain text representation of k-mer sets
Source: Genome Biol. 2023 Jun 9;24:136. doi: 10.1186/s13059-023-02968-z (PMC10251615; doi:10.1186/s13059-023-02968-z)
Supplement: Supplementary file 1 — Additional file 1. Additional experiments and proof of optimality. Additional CL and SC data from our experiments, with varying k and min abundance. Also, the average unitig length and total unitig count is plotted. Performance measurements with varying amount of threads. A query experiment with Bifrost as well as an SSHash-Lite experiment on a machine with focus on single-core performance. Proof of optimality for our algorithm. [file 13059_2023_2968_MOESM1_ESM.pdf]

1 Additional figures

Figure S1: Performance with different amounts of threads

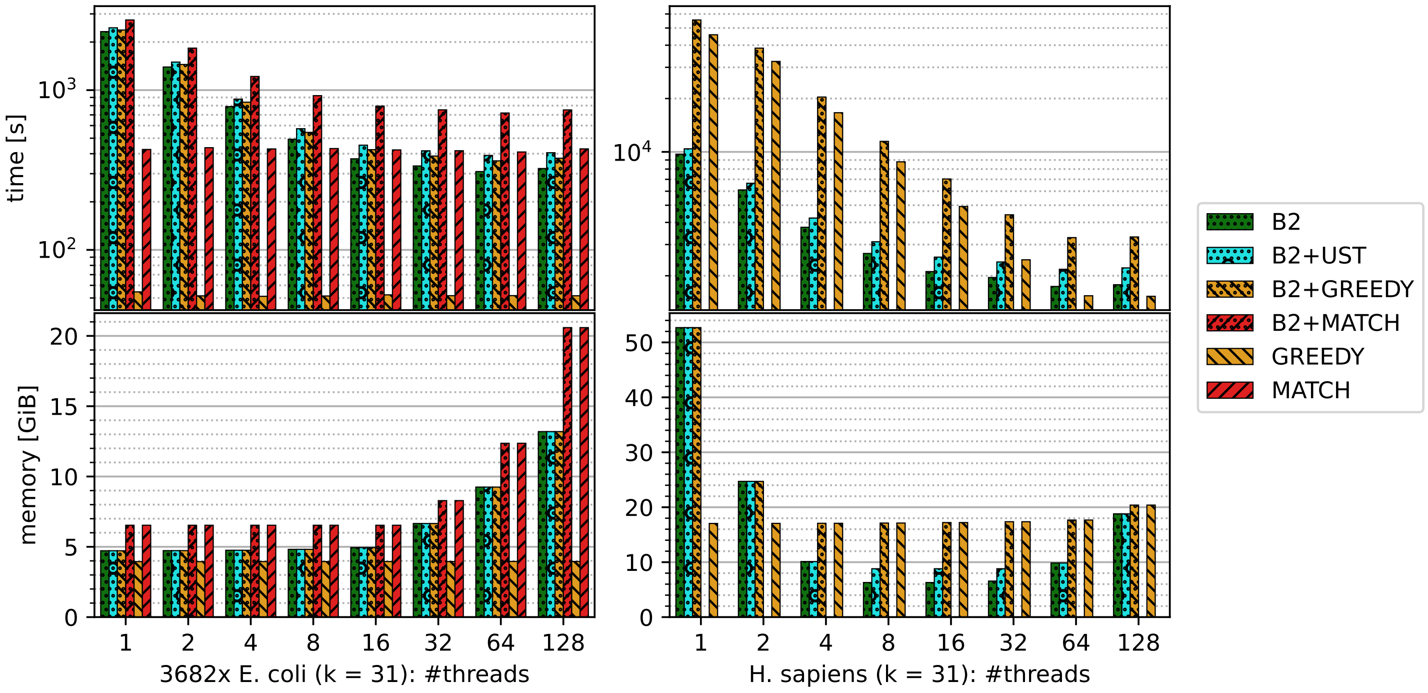

Time and memory consumption of different compression methods for the Escherichia coli pangenome and the Homo sapiens genome. We chose a min abundance of 1 and varied the number of threads. Since UST cannot be run with more than one thread, it is always run with one thread (the preceding run of BCALM2 was executed with the correct number of threads). For UST, greedy matchtigs and matchtigs, note that they take unitigs as input, so they require a run of BCALM2 as preprocessing. The time in these cases is given as the sum of the time taken by both tools, and the memory as the maximum.

Figure S2: Quality of compressing model organisms

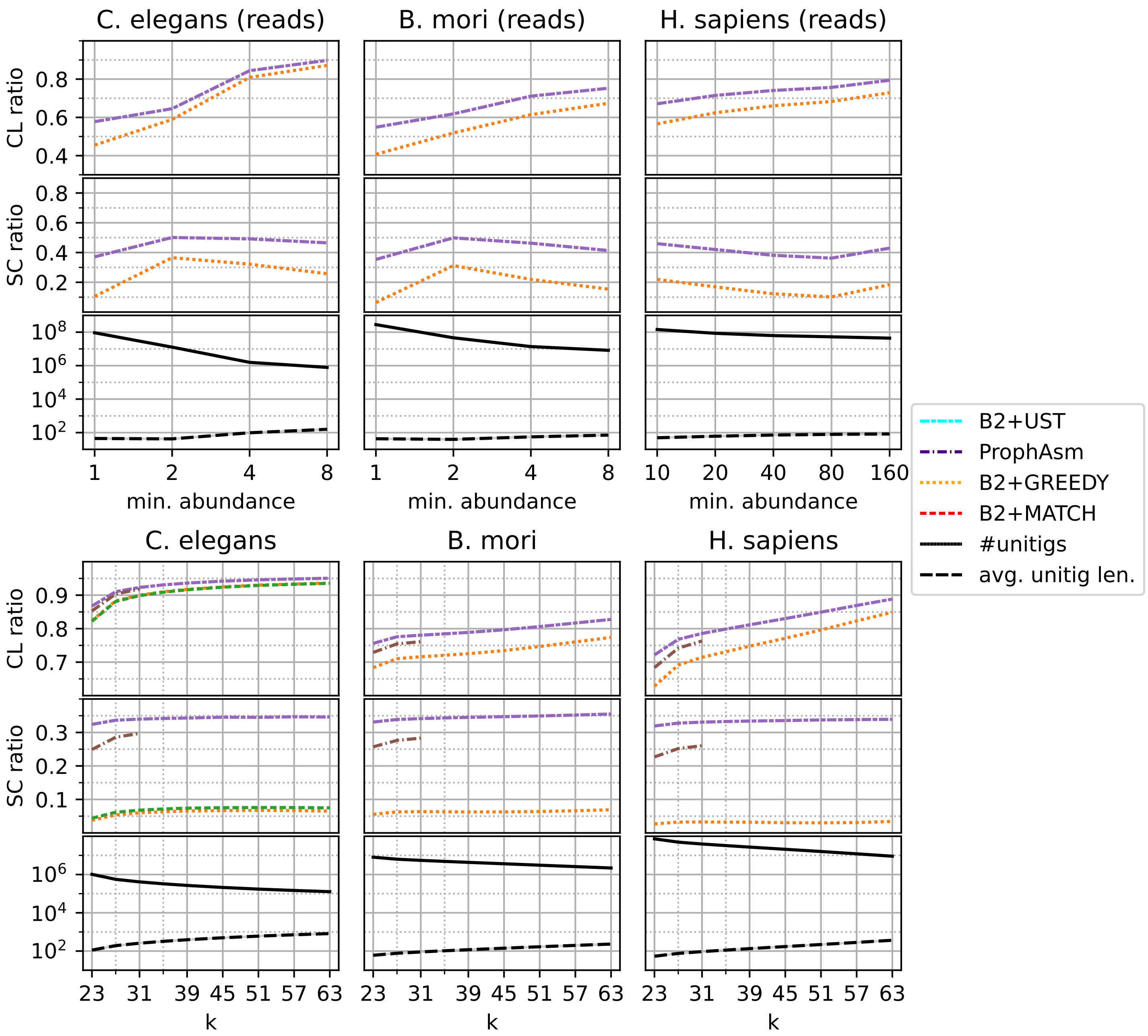

Ratio of CL and SC between different compression methods and unitigs, as well as the number and average length of unitigs for reference genomes and short reads of model organisms. For read data sets we chose $k = 51$, and for the reference genomes we chose a min abundance of 1. ProphAsm handles only $k \leq 32$ and matchtigs are memory-feasible only for C. elegans, so only the corresponding runs are shown. ProphAsm and UST produce overlapping lines in all subplots, and matchtigs and greedy matchtigs mostly overlap. The lengths of the genomes are 100Mbp for Caenorhabditis elegans, 482Mbp for Bombyx mori and 3.21Gbp for Homo sapiens and the read data sets have a coverage of 64x for Caenorhabditis elegans, 58x for Bombyx mori and 300x for Homo sapiens.

Figure S3: Performance of compressing model organisms

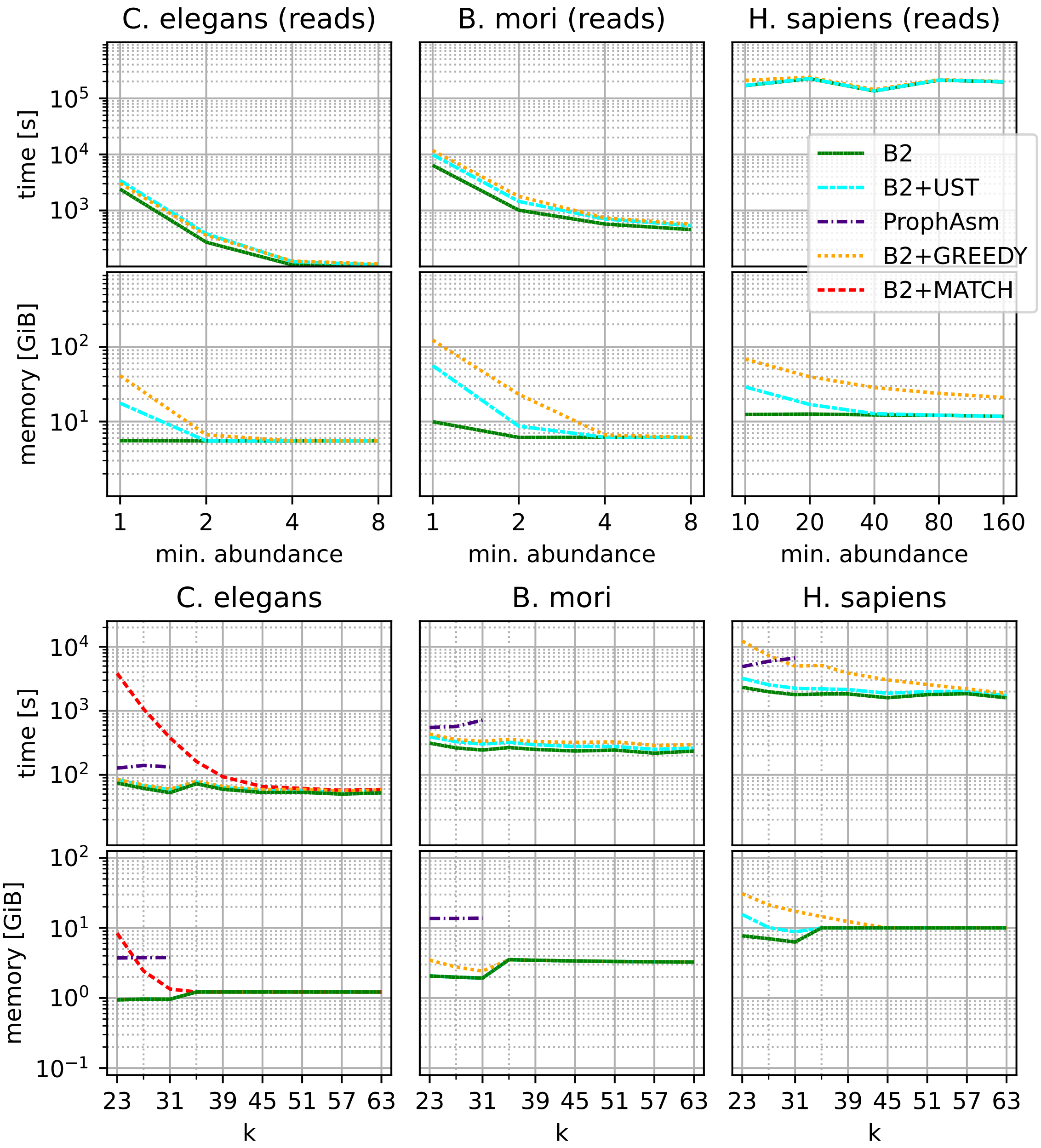

Time and memory consumption of different compression methods for reference genomes and short reads of model organisms. The read data sets are processed with $k = 51$ and varying min abundance, and the reference genomes with a min abundance of 1 and varying k. The time and memory of algorithms including BCALM2 overlap often, since they are dominated by BCALM2. BCALM2, greedy matchtigs and matchtigs are run with 28 threads, while UST and ProphAsm can only be run with one thread (for UST, the preceding run of BCALM2 was executed with 28 threads). For UST, greedy matchtigs and matchtigs, note that they take unitigs as input, so they require a run of BCALM2 as preprocessing. The time in these cases is given as the sum of the time taken by both tools, and the memory as the maximum. ProphAsm cannot handle $k > 32$ and matchtigs run out of memory for all but C. elegans, so the corresponding runs are not shown.

Figure S4: Quality of compressing pangenomes

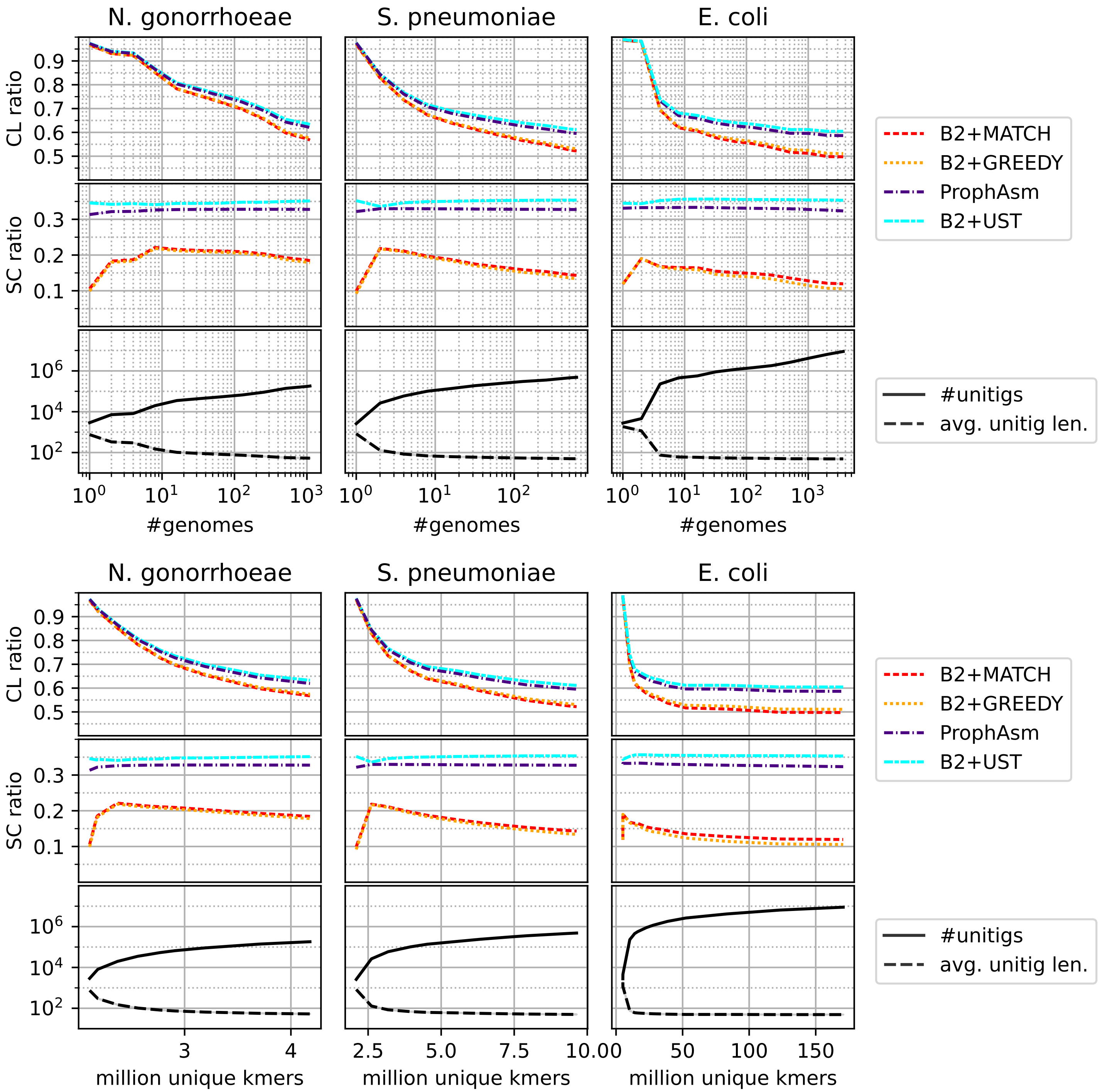

Ratio of CL and SC between different compression methods and unitigs, as well as the number and average length of unitigs for pangenomes. We chose $k = 31$, and a min abundance of 1. ProphAsm and UST produce overlapping lines in all subplots, and matchtigs and greedy matchtigs mostly overlap. The lengths of the genomes are 2.15Mbp for Neisseria gonorrhoeae, 2.22Mbp for Streptococcus pneumoniae and 4.64Mbp for Escherichia coli.

Figure S5: Performance of compressing pangenomes

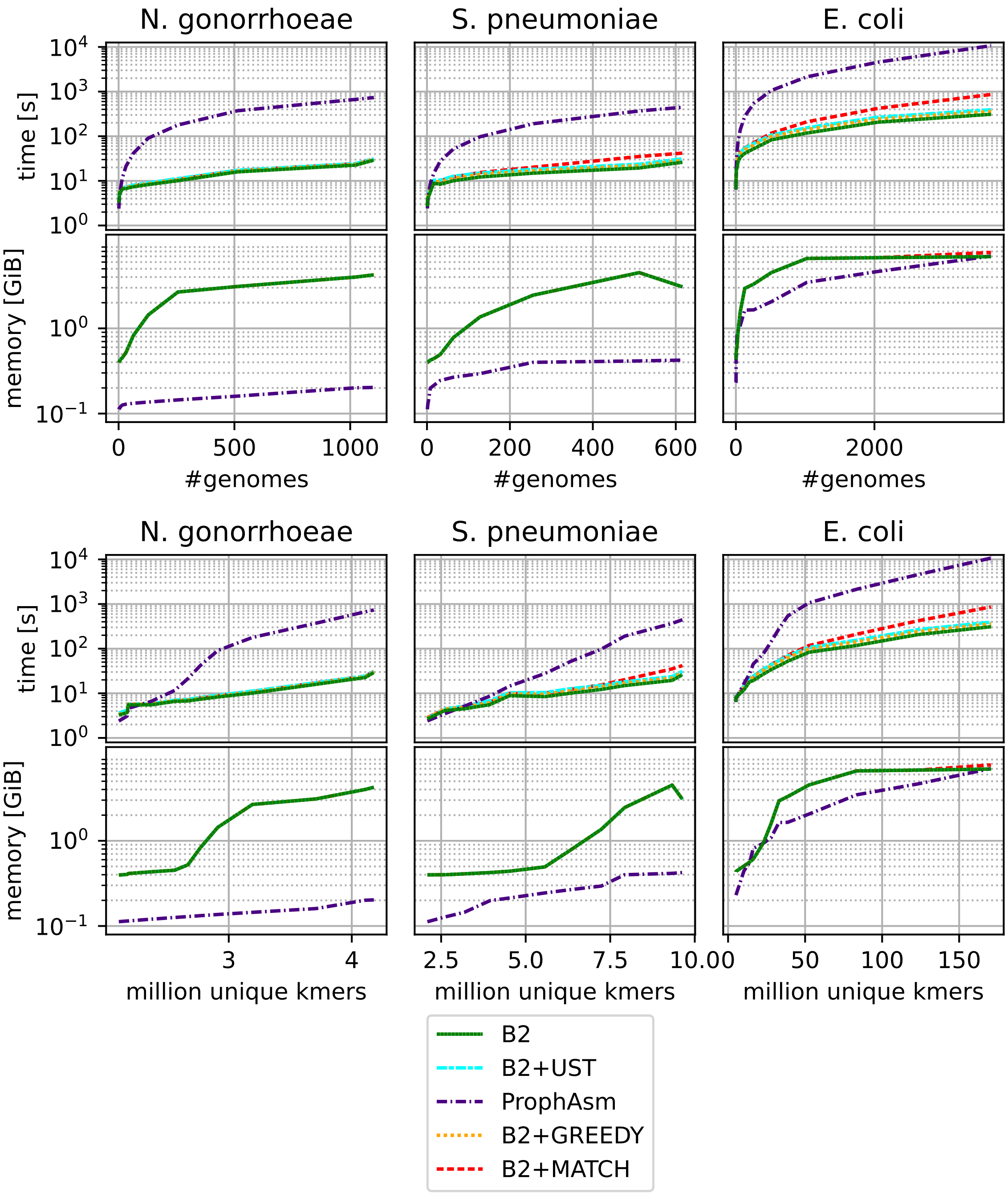

Time and memory consumption of different compression methods for pangenomes. We chose $k = 31$ and a min abundance of 1. The time and memory of algorithms including BCALM2 overlap often, since they are dominated by BCALM2. BCALM2, greedy matchtigs and matchtigs are run with 28 threads, while UST and ProphAsm can only be run with one thread (for UST, the preceding run of BCALM2 was executed with 28 threads). For UST, greedy matchtigs and matchtigs, note that they take unitigs as input, so they require a run of BCALM2 as preprocessing. The time in these cases is given as the sum of the time taken by both tools, and the memory as the maximum.

2 Query experiment on a machine with focus on single-core performance

Table S1: Performance characteristics of querying different tigs with SSHash-Lite.

(a) regular SSHash-Lite

genome	algorithm	index time [min]	search time [sec]	search speedup	index size [GiB]	size imprv.
~309kx	unitigs	1.86	1084	1.00	0.99	1.00
Salmonella	UST	1.38	568	1.91	0.67	1.48
(0.75)	gMatchtigs	1.48	339	3.20 (1.68)	0.62	1.60 (1.08)
Human	unitigs	13.8	274	1.00	4.39	1.00
reads	UST	11.4	252	1.10	3.46	1.27
(0.75)	gMatchtigs	12.9	208	1.31 (1.2)	3.31	1.33 (1.05)
2505x	unitigs	9.50	249	1.00	3.57	1.00
Human	ProphAsm	8.58	215	1.16	2.84	1.26
(0.65)	gMatchtigs	9.02	188	1.32 (1.14)	2.84	1.26 (1.00)

(b) canonical SSHash-Lite

genome	algorithm	index time [min]	search time [sec]	search speedup	index size [GiB]	size imprv.
~309kx	unitigs	2.81	413	1.00	1.08	1.00
Salmonella	UST	2.11	286	1.44	0.75	1.44
(0.75)	gMatchtigs	2.36	211	1.96 (1.36)	0.71	1.52 (1.06)
Human	unitigs	19.5	156	1.00	4.79	1.00
reads	UST	16.3	145	1.08	3.86	1.24
(0.75)	gMatchtigs	17.7	129	1.18 (1.09)	3.76	1.27 (1.03)
2505x	unitigs	12.0	134	1.00	3.93	1.00
Human	ProphAsm	13.1	122	1.10	3.19	1.23
(0.65)	gMatchtigs	13.2	114	1.18 (1.07)	3.22	1.22 (0.99)

SSHash-Lite is run with $k = 31$ and a kmer-inclusion rate of 0.8. On the Salmonella pan-genome we used a minimizer length of 17 for the regular index and a minimizer length of 16 for the canonical index. On the human reads we used a minimizer length of 20 for the regular index and a minimizer length of 19 for the canonical index. On the human pangenome we used a minimizer length of 19 for the regular index and a minimizer length of 20 for the canonical index. The search speedup is with respect to unitigs, and the search speedup in parentheses is with respect to the strings computed by UST. Index time is the end-to-end time required to build the SSHash-Lite index: it includes reading the collections from disk and building the data structure using external memory. Searching time is the time required to check which reads have at least 80% of their kmers in the input SPSS. The number in parentheses under the genome is the kmer hitrate, i.e. the fraction of kmers from the query that are part of the queried dataset. These experiments were performed on an Intel Core i9-9900K CPU, clocked at 3.60 GHz and the code was compiled with gcc 11.2.

3 Query experiment on E. coli with Bifrost

Matchtigs have further applications beyond merely reducing the size required to store a set of kmers. Due to their smaller size and lower string count, they can make downstream applications more efficient. For example, the kmer-based query tool Bifrost [23]achieves speedups of 1.66 when using matchtigs instead of unitigs, and 1.29 when using matchtigs instead of the strings computed by ProphAsm (see Table S2 for the detailed results). Note that we measure the speedup of the search phase only, since the index phase is independent of the query and therefore could be moved into a separate preprocessing step.

Table S2: Performance characteristics of querying different tigs with Bifrost.

genome	algorithm	indexing [s]	searching [s]	total [s]	search speedup		mem [GiB]
3682x E. coli	unitigs	21.00	336.79	360.37	1.00		2.39
	UST	16.11	267.02	285.52	1.26		2.13
	ProphAsm	15.55	261.65	278.91	1.29		2.10
	gMatchtigs	16.19	208.10	226.23	1.62	(1.26)	2.12
	matchtigs	16.33	202.71	220.73	1.66	(1.29)	2.09

The Bifrost query command is run with 16 threads and default settings. 3682 E.coli genomes are queried with all raw reads used to assemble 30 of the genomes. All columns are averages of 10 experiments. The search speedup is with respect to unitigs, and the search speedup in parentheses is with respect to the strings computed by ProphAsm. Indexing is the time required to build the minimizer index within Bifrost, and searching is the time required to check if a read is in the pangenome using the minimizer index. The total time is the wall-clock time of bifrost.

Note also that these speedups are consistent with those reported when using heuristic simplitigs [43]for kmer queries with BWA-MEM [46]without modification of BWA-MEM).

We achieve our speedups *without modifying Bifrost*[1], but just by passing Bifrost a file with our tigs instead of unitigs. When using in query mode, Bifrost is intended to take a GFA file containing a compacted de Bruijn graph as well as a file containing the query strings as inputs. It then builds a minimizer index over the unitigs in the GFA file, and executes the queries using this index. If we pass a GFA file containing e.g. matchtigs instead of a compacted de Bruijn graph, then Bifrost still works the same way. It builds a minimizer index over the matchtigs inside the GFA file, and then uses that index to execute the queries. It does not build a compacted de Bruijn graph from the matchtigs, or in any other way use any topology information that would be in the GFA file. This is why the query still works correctly, even though the GFA file does not contain a compacted de Bruijn graph.

The speedups that we achieve when passing e.g. matchtigs instead of unitigs in the GFA file can be explained as follows. When querying, Bifrost iterates over the kmers of the query. It finds the tig containing the kmer using the minimizer index and then extends that match until the query and the tig differ, or the query or tig ends. In that case, it again queries the minimizer index, until all query kmers are checked. When using longer tigs with a lower tig count, then the number of queries to the minimizer index is lower, as the end of a tig is reached more rarely. Extending a match is extremely fast, as it only requires a comparison between the character following the last match on the tig and the character following the last match on the query. However querying the minimizer index requires to compute the hash of the current minimizer, as well as looking up the hash inside the hash table, and then checking which tig does actually contain the original kmer, and not just the minimizer. Therefore, by reducing the number of queries to the minimizer index, we can reduce the overall runtime of the query.

In addition to speedups, a smaller representation is also expected to decrease the memory consumption. However in practice the memory consumption only increases when switching from unitigs to heuristic simplitigs, but not when further switching to matchtigs or greedy matchtigs. We assume that this is due to the longer strings requiring more memory while being loaded block-wise in ASCII format before they get stored in a two-bits-per-character compressed format.

4 Proofs of optimality

In this subsection we provide proof for the minimality of the SPSS computed by our algorithm. We call the input set of strings I, and the output SPSS S. We assume that the graph $DBG_k(I)$ is connected. If it is not, then executing our algorithm on each maximal connected subgraph yields an optimal solution. This is because there can only be breaking arcs between different maximal connected subgraphs, i.e. every string in S is within only a single maximal connected subgraph. Therefore, processing the maximal connected subgraphs separately does not decrease the solution space.

We recite some definitions from the main matter for convenience.

Definition 1 (Graph transformation)**.** *Given an arc-centric de-Bruijn graph $DBG_k(I) = (V, E)$, the* transformed graph *is defined as $DBG'_k(I) = (V, E')$ where E' is a multiset defined as $E' := E \cup (V \times V)$. In E', arcs from E are marked as*

[1] We did need to add code to measure index and search time separately.

non-breaking*, and arcs from* $V \times V$ *are marked as* breaking *arcs. The* cost function $c(e), e \in E'$ *assigns all non-breaking arcs the costs* 1 *and all breaking arcs the costs* $k-1$.

Definition 2 (Circular original-biarc-covering biwalk)**.** *Given a transformed graph* $DBG'_k(I) = (V, E)$*, a* circular original-biarc-covering biwalk *is a circular biwalk* w *such that for each non-breaking arc* $(a, b) \in E$ *there is a biarc* $[(a, b), (b^{-1}, a^{-1})]$, $[(b^{-1}, a^{-1}), (a, b)]$ *or* $[(a, b)]$ *in* w*. Additionally,* w *needs to contain at least one breaking biarc.*

Definition 3 (Costs of a circular original-biarc-covering biwalk)**.** *Given a transformed graph* $DBG'_k(I)$ *and a circular original-biarc-covering walk* w *possibly consisting of biarcs* $[(a, b), (b^{-1}, a^{-1})]$ *and self-complemental biarcs* $[(a, b)]$*. The* costs $c(w)$ *of* w *are the sum of the costs of each biarc and self-complemental biarc, where the costs of a biarc* $[(a, b), (b^{-1}, a^{-1})]$ *are* $c((a, b))$*, and the costs of a self-complemental biarc* $[(a, b)]$ *are* $c((a, b))$*.*

4.1 Reduction to the bidirected partial-coverage Chinese postman problem.

First, we show that the reduction to the bidirected partial-coverage Chinese postman problem is correct. For that we assume that we are given a solver for this variant of the Chinese postman problem, and formally recite our algorithm as follows:

Algorithm 4 (Chinese-postman-based algorithm)**.**

1. *Compute the arc-centric de Bruijn graph* $DBG_k(I)$*.*
2. *Compute the transformed graph* $DBG'_k(I)$ *(Definition 1).*
3. *Use the given solver to compute a circular original-biarc-covering biwalk* w^* *of minimum cost (Definition 2).*
4. *Break* w^* *at all breaking biarcs.*
5. *Output set of strings* S *spelled by the resulting walks.*

We first show that the algorithm only outputs SPSS and that the size of the final SPSS is equal to the costs of the biwalk w^* computed in the algorithm. Using these lemmas, we can finally prove that the SPSS output by the algorithm is minimum.

Lemma 5 (The Chinese-postman-based algorithm outputs an SPSS)**.** *When applying Steps 4 and 5 of Algorithm 4 to a circular original-biarc-covering biwalk* w *in a transformed graph* $DBG'_k(I)$*, then the result is an SPSS of* I*.*

Proof. Observe that w contains each non-breaking biarc of $DBG'_k(I)$ by definition. When being broken in Step 4, only breaking biarcs are removed and hence only biarcs that are not connected to any input kmer. So, the broken biwalks contain all biarcs of $DBG_k(I)$. Furthermore, all breaking biarcs are removed, and the remaining biarcs all stem from $DBG_k(I)$. So, the broken biwalks contain only biarcs of $DBG_k(I)$, and therefore the same set of biarcs as $DBG_k(I)$. Therefore, the strings spelled in step 5 of Algorithm 4 contain the same kmers as the input (but they may be reverse complemented), and hence have the same spectrum. Concluding, S is an SPSS of I. □

Lemma 6 (Costs equal size)**.** *When applying Steps 4 and 5 of Algorithm 4 to a circular original-biarc-covering biwalk* w *in a transformed graph* $DBG'_k(I)$*, the resulting SPSS* S *has a size equal to the costs of* w*.*

Proof. We denote the result of breaking w at all breaking biarcs as W. Each walk $w' \in W$ spells a string $s' \in S$ and it holds that $|w'| + (k-1) = |s'|$. Further, w contains a breaking biarc for each walk in W, and we have assigned costs $k-1$ to each breaking biarc, and costs of 1 to each other biarc. Therefore, the costs of w equal the size of S. □

Lemma 7 (Optimality of the Chinese-postman-based algorithm)**.** *The set of strings* S *output by Algorithm 4 is a minimum SPSS of* I*.*

Proof. Algorithm 4 computes a minimum-cost circular original-biarc-covering biwalk w^* in the transformed graph $DBG'_k(I)$. It computes a set of strings S from w^* by applying steps 4 and 5 where S is an SPSS of I by Lemma 5. By Lemma 6, the costs of any minimum-cost circular original-biarc-covering biwalk w' are equal to the size of the SPSS S' that is produced by applying Steps 4 and 5 to w'. So, since each SPSS can be represented by some w', the existence of an SPSS smaller than S would imply the existence of a minimum-cost circular original-biarc-covering biwalk cheaper than w^*, which contradicts the minimality of w^*. Therefore, S is of minimum size. □

4.2 Solving the bidirected partial-coverage Chinese postman problem with min-cost integer flows.

Given that our algorithm is correct if we feed it a correct minimum-cost circular original-biarc-covering biwalk, we need to show that we actually do. For that, we start by showing that a solution to the flow formulation in Section 5.5 correctly results in a minimum biEulerisation which results in a minimum-cost circular original-biarc-covering biwalk as required. We restate the flow formulation and all defined variables for the reader's convenience:

Definition 8 (Flow formulation)**.** *Given a transformed graph $DBG'_k(I) = (V, E)$, we define*

- *F to be the non-breaking arcs in E,*
- *$c_e := 1$ for $e \in F$ and $c_e := k-1$ for $e \in (E \setminus F)$, and*
- *S to be the set of all self-complemental nodes and $T := (V \setminus S)$.*

Then the flow formulation *of the bidirected partial-coverage Chinese postman problem in $DBG'_k(I)$ is:*

$$\min \quad \sum_{e \in E,\ e \text{ is canonical}} c_e x_e \ \text{s.t.} \tag{1}$$

$$x_e \text{ are non-negative integers, and} \tag{2}$$

$$\sum_{e \in (E \setminus F)} x_e \geq 1, \text{ and} \tag{3}$$

$$\forall v \in T: \quad \sum_{e \in E^-(v)} x_e \left(1 + \mathbb{1}_{e=e^{-1}}\right) - \sum_{e \in E^+(v)} x_e \left(1 + \mathbb{1}_{e=e^{-1}}\right)$$
$$= d_F^+(v) - d_F^-(v) + \left(\mathbb{1}_{(v,v^{-1}) \in F^+(v)} - \mathbb{1}_{(v^{-1},v) \in F^-(v)}\right), \text{ and} \tag{4}$$

$$\forall v \in S: \quad \left(d_F^+(v) + \sum_{e \in E^+(v)} x_e\right) \bmod 2 = 0, \text{ and} \tag{5}$$

$$\forall e \in E: \quad x_e = x_{e^{-1}} \tag{6}$$

Additionally, we formalise this part of our algorithm:

Algorithm 9.

1. *Compute the arc-centric de Bruijn graph $DBG_k(I)$.*
2. *Compute the transformed graph $DBG'_k(I)$ (Definition 1).*
3. *Build and solve the flow problem according to Definition 8.*
4. *Insert x_e copies of each arc $e \in E$ with flow value $x_e \geq 1$, and set the multiplicity of breaking arcs e to x_e (removing breaking arcs e with a flow value $x_e = 0$).*
5. *Compute a biEulerian circuit (see e.g. [47]for details).*

The final biEulerian circuit is the minimum-cost circular original-biarc-covering biwalk of $DBG'_k(I)$. To show that this is true, in the same pattern as above we first show that the algorithm only outputs valid circular original-biarc-covering biwalks, and then show that the costs that it optimises are equivalent to the costs of the output walk.

we first prove that the costs optimised by the flow problem can be transformed into the costs in the Chinese postman problem by addition of a constant. Then we show that for each (minimum) solution to the Chinese postman problem w, there exists a solution x to the flow instance that produces a solution w' to the Chinese postman problem if fed into steps 4 and 5 of Algorithm 9, where the costs $c(w)$ of w are equal to the costs $c(w')$ of w'. This is enough to argue that Algorithm 9 produces a minimum-cost circular original-biarc-covering biwalk of $DBG'_k(I)$.

Lemma 10. *Algorithm 9 computes a circular original-biarc-covering biwalk of $DBG'_k(I)$.*

Proof. We need to prove that given a solution to the flow formulation, the graph gets correctly biEulerised such that it admits a biEulerian circuit after step 4.

First, consider that due to Equation (2), the biEulerisation itself is well-defined, as there are only whole-numbered amounts of arcs to be added, and there are no negative numbers implying removal of arcs. Further, it holds that the biEulerised graph is a valid bigraph, since Equation (6) ensures that whenever an arc gets added, the corresponding reverse complement gets added as well.

Next, we show that Equations (4) and (5) ensure that the flow results in a valid biEulerisation, i.e. that the bi-imbalance of each node is zero after applying the biEulerisation. We define x'_e be the multiplicity of each arc after applying the biEulerisation, defined as $x'_e := x_e + 1$ for non-breaking arcs and $x'_e := x_e$ for breaking arcs.

- If v is not self-complemental, then it is constrained by Equation (4). We can rewrite it as follows:

$$\sum_{e \in E^-(v)} x'_e \left(1 + \mathbb{1}_{e=e^{-1}}\right) = \sum_{e \in E^+(v)} x'_e \left(1 + \mathbb{1}_{e=e^{-1}}\right). \tag{7}$$

  And, using d'^+ and d'^- to denote degrees in the biEulerised graph as well as $\in_\#$ to denote the operator that counts the number of occurrences of an element in a multiset, we get:

$$0 = d'^+(v) - d'^-(v) + (((v, v^{-1}) \in_\# E^+(v)) - ((v^{-1}, v) \in_\# E^-(v))).$$

  The right part of the equation is the generalised formula for the bi-imbalance, allowing for duplicate self-complemental arcs. Since it is zero, it holds that the bi-imbalance of v is zero.

- If v is self-complemental, then it is constrained by Equation (5). We can rewrite it as follows:

$$\left(\sum_{e \in E^+(v)} x'_e\right) \bmod 2 = 0. \tag{8}$$

  Using d'^+ to denote the out-degree in the biEulerised graph, we get:

$$d'^+(v) \bmod 2 = 0. \tag{9}$$

  The right part of the equation is the formula for the bi-imbalace for self-complemental nodes. Since it is zero, it holds that the bi-imbalance of v is zero.

Since the bi-imbalance of all nodes is zero, by definition of the bi-imbalance, the biEulerised graph admits a biEulerian circuit. This is computed and output in step 5. The result is a circular original-biarc-covering biwalk, because by Equation (3), the biEulerisation and therefore also the final graph contain a breaking arc. □

Lemma 11 (Chinese postman costs equal flow costs)**.** *Let $DBG'_k(I)$ be the transformed graph of I. Let x be a solution to the flow formulation of $DBG'_k(I)$. Let w be a minimum-cost circular original-biarc-covering biwalk of $DBG'_k(I)$. If each non-breaking arc $e \in F$ appears in $x_e + 1$ biarcs in w, and each breaking arc $e \in (E \setminus F)$ appears in x_e biarcs in w, then $c(w) = |F| + \sum_{e \in E,\ e \text{ is canonical}} c_e x_e$.*

Proof. The costs $c(e)$ of arcs e in the circular original-biarc-covering biwalk are equal to the costs of arcs c_e in the flow formulation. Additionally, for each biarc $[e, e^{-1}]$, by definition it holds that $c_e = c_{e^{-1}}$.

Let $e \in E$ be an arc and $e^{-1} \in E$ its reverse complement. Since only one of them is canonical (or they are equal), e and e^{-1} together contribute $c_e \cdot x_e$ in the flow formulation.

On the other hand, since e appears in $x_e + 1$ (or x_e) biarcs in w, it holds that e^{-1} appears in the same $x_e + 1$ (or x_e) biarcs in w. Therefore, together they contribute $(x_e + 1) \cdot c_e$ to the costs of w if they are non-breaking, and $x_e \cdot c_e$ if they are breaking. If they are non-breaking, it holds that $c_e = 1$, and therefore $(x_e + 1) \cdot c_e = x_e \cdot c_e + 1$. Resulting, since there are $|F|$ non-breaking arcs, it holds that $c(w) = |F| + \sum_{e \in E,\ e \text{ is canonical}} c_e x_e$. □

Lemma 12 (Min-cost flow can reproduce solutions at the same costs)**.** *Given a circular original-biarc-covering biwalk w in a transformed graph $DBG'_k(I)$, there exists a solution x to the flow formulation of $DBG'_k(I)$ that produces a circular original-biarc-covering biwalk w' if fed into steps 4 and 5 of Algorithm 9, where the $c(w) = c(w')$.*

Proof. We construct x by setting x_e to the number of occurrences of arc e in w, and then subtracting one from each x_e if $e \in F$. Then, by Lemma 11, it holds that $c(w) - |F|$ equals the costs of x. Further, the circular original-biarc-covering biwalk w' produced by 4 and 5 of Algorithm 9 contains x_e occurrences of each arc $e \in E$, plus one additional occurrence of each non-breaking arc $e \in F$. Since each arc in $e \in F$ has costs $c_e = 1$, it holds that $c(w') - |F|$ equals the costs of x, so $c(w) = c(w')$.

It remains to show that x is a valid solution to the flow formulation. We show that each condition holds.

(2) Holds.

(3) By definition, w contains at least one breaking biarc, which by definition is an arc $e \in (E \setminus F)$. For this e it holds that $x_e \geq 1$.

(4) Let $v \in V$ be a node that is not self-complemental. We can rewrite Equation (4) as follows:

$$\begin{aligned} &d_F^-(v) + \mathbb{1}_{(v^{-1},v)\in F^-(v)} + \sum_{e\in E^-(v)} x_e \left(1 + \mathbb{1}_{e=e^{-1}}\right) \\ &= d_F^+(v) + \mathbb{1}_{(v,v^{-1})\in F^+(v)} + \sum_{e\in E^+(v)} x_e \left(1 + \mathbb{1}_{e=e^{-1}}\right) \end{aligned}$$

By defining $x'_e := x_e$ for breaking arcs and $x'_e := x_e + 1$ for non-breaking arcs, we can further rewrite the equation:

$$\sum_{e\in E^-(v)} x'_e \left(1 + \mathbb{1}_{e=e^{-1}}\right) = \sum_{e\in E^+(v)} x'_e \left(1 + \mathbb{1}_{e=e^{-1}}\right) \tag{10}$$

By definition, x'_e is the number of occurrences of arc e in w. We decompose $w = ([e_1, e_1^{-1}], \ldots, [e_{|w|}, e_{|w|}^{-1}])$ into two circular walks w_1, w_2 where $w_1 := (e_1, \ldots, e_{|w|})$ and $w_2 := (e_{|w|}^{-1}, \ldots, e_1^{-1})$. The number of occurrences of each arc in w is the same as the sum of occurrences in w_1 and w_2, except for self-complemental arcs which occur twice as often in w_1 and w_2. Therefore, Equation (10) counts the sum of arcs in w_1 and w_2 that enter v on the left side, and the sum of arcs that leave v on the right side. Since w_1 and w_2 are circular walks, this implies that the sums are equal.

(5) Let $v \in V$ be a self-complemental node. By defining $x'_e := x_e$ for breaking arcs and $x'_e := x_e + 1$ for non-breaking arcs, we can rewrite Equation (5) as follows:

$$\left(\sum_{e\in E^+(v)} x'_e\right) \bmod 2 = 0 \tag{11}$$

By definition, x'_e is the number of occurrences of arc e in w. Since v is self-complemental, it can only be entered or left via arcs that are not self-complemental. Therefore, whenever w contains v, it contains one arc that leaves v, and another arc that leaves v^{-1} in its reverse complement. Since $v = v^{-1}$, each element of the sum in Equation (11) is even, so the whole sum is even, so the equation holds.

(6) If $e \in E$ is self-complemental, this condition is a tautology. If not, then each biarc in w that contains e also contains e^{-1}, so it holds that $x_e = x_{e^{-1}}$. □

Lemma 13. *Algorithm 9 computes a minimum-cost circular original-biarc-covering biwalk w of $DBG'_k(I)$.*

Proof. By Lemma 12 it holds that for a minimum-cost circular original-biarc-covering biwalk w', there exists a flow solution that produces another (or the same) minimum-cost circular original-biarc-covering biwalk w. By Lemma 10, it holds that Algorithm 9 computes a circular original-biarc-covering biwalk of $DBG'_k(I)$, so it cannot compute any walk of lower costs, otherwise w' would not have been minimum-cost. □

From Lemma 13 it follows that we can use the flow formulation with Algorithm 9 to solve step 3 of Algorithm 4 and get a complete algorithm to compute matchtigs.

4.3 Solving the min-cost integer flow formulation with min-cost matching.

As argued above, the flow formulation is not actually practically useful, therefore we solve it via min-cost perfect matching. For this, we are given the set of min-cost paths between unbalanced nodes as follows:

Definition 14 (Matching paths)**.** *Let $DBG_k(I)$ be an arc-centric de Bruijn graph. Let S be the set of its self-complemental nodes with positive bi-imbalance, A be the set of its nodes with negative bi-imbalance and B be the set of its nodes with positive bi-imbalance that are not self-complemental. We define $P := \{(u, v, c(u, v)) \mid u \in A \cup S \wedge v \in B \cup S \wedge c(u, v) \leq k-1\}$ as the* set of min-cost source-sink paths *in $DBG_k(I)$, where $c(u, v)$ are the costs of the min-cost path from u to v where all arcs have costs of one. We additionally define $P_{u,v} := \min\{(\{u, v\}, c) \mid \exists u' \in \{u, u^{-1}\}, v' \in \{v, v^{-1}\} : (\{u', v'\}, c) \in P \vee (\{v', u'\}, c) \in P\}$ as the minimum min-cost path between u and v or their reverse complements, where min-cost paths are compared by their costs c. If there are no min-cost paths between u and v (i.e. the minimum is taken from the empty set), then $P_{u,v} := \perp$.*

We informally describe the matching instance in Section 5.6, so we formally restate it here for the purpose of our proof.

Definition 15 (Matching instance)**.** *Let $DBG_k(I)$ be an arc-centric de Bruijn graph. Let S be the set of its self-complemental nodes with positive bi-imbalance, A be the set of its nodes with negative bi-imbalance and B be the set of its nodes with positive bi-imbalance that are not self-complemental.*

Then the matching graph is defined as:

$$\begin{aligned} M &= (V, E) \\ V &= V_1 \cup V_2 \\ V_1 &= \{v_i+, v_i- \mid v \in (A \cup B \cup S) \wedge v \textit{ is canonical} \wedge i \in \{1, \ldots, |bi_v|\}\} \\ V_2 &= \{u+, u-, w+, w-\} \quad \texttt{// extra nodes for Equation (3)} \\ E &= E_1 \cup E_2 \cup E_3 \end{aligned}$$

where

- $E_1 = \{(\{v_i+, v_i-\}, k-1) \mid v_i+ \in V_1\}$ *are the edges that allow nodes to stay "unmatched" (note that condition $v_i+ \in V_1$ is equivalent to $v_i- \in V_1$ by definition of V_1),*
- $E_2 = \{(\{v_i+, v'_j+\}, c), (\{v_i-, v'_j-\}, c) \mid (\{v, v'\}, c) = P_{v,v'} \wedge v_i+, v'_j+ \in V_1\}$ *are the edges for sending flow between v and v' to balance them, and*
- $E_3 = \bigcup_{v_i+ \in V_1} \{(\{u+, v_i+\}, 0), (\{w+, v_i+\}, 0), (\{u-, v_i-\}, 0), (\{w-, v_i-\}, 0)\}$ *are the edges allowing one pair of nodes to be connected via a breaking biarc for free.*

We use the matching instance to solve the flow formulation as follows:

Algorithm 16.

1. *Compute the arc-centric de Bruijn graph $DBG_k(I)$.*
2. *Compute the set of min-cost source-sink paths P in $DBG_k(I)$.*
3. *Build and solve the matching instance $M = (V_1 \cup V_2, E_1 \cup E_2 \cup E_3)$ using a min-cost perfect matching algorithm.*
4. *Compute the transformed graph $DBG'_k(I) = (V, E)$.*
5. *Initialise the solution to the flow formulation with $x_e = 0$ for all $e \in E$.*
6. *For each arc $p \in E_2$ part of the matching solution, and for each min-cost path in P represented by p, increment the flow values x_e of all arcs $e \in E$ that are on a min-cost path by one.*

For showing that this algorithm computes a minimum-cost flow, we show that valid (not necessarily minimum) perfect matchings and (not necessarily minimum) flows are equivalent in terms of costs. But perfect matchings actually just represent a subset of possible flows. However, if a flow cannot directly be transformed into a matching, it is not minimum.

Definition 17 (Path-minimum flow)**.** *A* path-minimum flow *is a not necessarily minimum flow in the flow formulation of a transformed graph $DBG'_k(I) = (V, E)$ such that the flow can be decomposed into a set of source-sink paths such that each path is a shortest path if each arc has weight one. A source is a node with negative imbalance or a self-complemental node with positive imbalance, and a sink is a node with positive imbalance.*

Lemma 18. *Each minimum flow is path-minimum.*

Proof. Let x be a minimum flow in the flow formulation. Equations (4) and (5) ensure that the flow can be decomposed into a set of source-sink paths. If any of these paths were not min-cost, it could be replaced with a min-cost path, resulting in a cheaper flow and contradicting the minimality of x. Therefore, x is path-minimum. □

Lemma 19. *Each path-minimum flow x of costs $c(x)$ can be transformed into a perfect matching E_M of costs $c(E_M) = 2(c(x) - (k-1))$.*

Proof. We construct the perfect matching E_M as follows:

- Pick some breaking arc e_b with positive flow (it exists because of Equation (3)) and add the edges $\{u+, v_1+\}$, $\{w+, v'_1+\}$, $\{u-, v_1-\}$ and $\{w-, v'_1-\}$ corresponding to e_b to E_M.
- For each breaking arc e, repeat the following x_e times, or $x_e - 1$ times if $e = e_b$: add the corresponding edges of the form $\{v_i+, v_i-\}$ to E_M, choosing an arbitrary i such that no node gets matched twice.
- For each source-sink biwalk p in the decomposed flow that contains no breaking biarcs, add the edges $\{v_i+, v'_j+\}$ and $\{v_i-, v'_j-\}$ corresponding to p to E_M. Choose an arbitrary i and j such that no node gets matched twice. Repeat this step according to the minimum flow value on any arc in p.

We show that E_M is a perfect matching. In the decomposition of x, each unbalanced binode is start or end of as many biwalks as there are copies of it in the matching instance. This specifically holds for walks from a binode to its complement binode, which alter the bi-imbalance of the corresponding binode by two, and also connect two nodes in the matching problem that correspond to the same binode. Since the matching instance has copies of each binode according to its absolute bi-imbalance, this means that all nodes in the matching instance are matched exactly once. Further, the arbitrarily picked arc e_b ensures that also $u+, u-, w+$ and $w-$ are matched. So, E_M is a perfect matching.

In E_M, pairs of edges correspond source-sink biwalks in x. In the flow formulation the costs of each biwalk are counted only in one direction, while in the matching instance, the costs are counted twice. Therefore, the costs of E_M are twice those of x. However, the nodes corresponding to the picked arc e_b are matched at no cost, whereas x contains a breaking arc of costs $k-1$ between them. Therefore, the costs of E_M are $c(E_M) = 2(c(x) - (k-1))$. □

Lemma 20. *Each perfect matching E_M of costs $c(E_M)$ can be transformed into a path-minimum flow x of costs $c(x) = c(E_M)/2 + (k-1)$.*

Proof. Note that each solution of the matching problem can be transformed into a solution of the matching problem which is symmetric, meaning that if an arc $\{v_i+, v'_j-\}$ is in E_M, then also $\{v_i-, v'_j+\}$ is in E_M, as well as if n arc $\{v_i+, v'_j+\}$ is in E_M, then also $\{v_i-, v'_j-\}$ is in E_M. Using this symmetry, we construct the path-minimum flow x as follows:

- For each matched edge of the form $\{v_i+, v'_j+\}$ and $\{v_i-, v'_j-\}$, we add a flow of one to the corresponding biwalk in x.
- For each pair of distinct matched edges of the form $\{v_i+, v_i-\}$, $\{v'_j+, v'_j-\}$ we add flow to the corresponding breaking biarc in x.
- For the matched edges $\{u+, v_1+\}, \{w+, v'_1+\}, \{u-, v_1-\}$ and $\{w-, v'_1-\}$ we add flow to the corresponding breaking biarc in x.

We show that x is a valid flow. Each unbalanced binode has copies in the matching instance according to its absolute bi-imbalance. Therefore, in the constructed flow x, source and sink nodes are balanced. Also, all other nodes are balanced, as they were balanced before, and are only passed via paths that keep them balanced. Therefore, x is a valid flow, and by construction it is path-minimum.

In E_M, pairs of edges correspond to source-sink biwalks in x. In x the costs of each biwalk are counted only in one direction, while in the matching instance, the costs are counted twice. Therefore, the costs of E_M are twice those of x. However, the nodes corresponding to the picked arc e_b are matched at no cost, whereas x contains a breaking arc of costs $k-1$ between them. Therefore, the costs of x are $c(x) = c(x)/2 + (k-1)$. □

Theorem 21. *The matchtigs algorithm composed of Algorithms 4, 9 and 16 is correct.*

Proof. Algorithm 4 is correct by Lemma 7. Algorithm 9 is correct by Lemma 13. Algorithm 16 computes a min-cost flow because minimum-cost flows are path-minimum by Lemma 18, and path-minimum flows are equivalent to perfect matchings in terms of costs by Lemmas 19 and 20. □
