# Supplementary material for: Matchtigs: minimum plain text representation of k-mer sets
Source: Genome Biol. 2023 Jun 9;24:136. doi: 10.1186/s13059-023-02968-z (PMC10251615; doi:10.1186/s13059-023-02968-z)
Supplement: Supplementary file 6 — Additional file 6. Review history. [file 13059_2023_2968_MOESM6_ESM.docx]

**1^st^ round**

**Reviewer 1**

Summary:

The authors introduce "Matchtigs", an alternative construct applicable for the representation of k-mer sets in plain text form. Their principal proposal is the design of a polynomial-time algorithm that solves a relaxed version of the originally proposed corresponding problem by Rahman and Medvedev 2021 [1].

The algorithm cleverly connects their proposed minimum plain-text k-mer set representation problem to the problem of Eulerising a graph. They reduce the minimum representation problem to that of graph Eulerisation. They cast it as a minimum-cost maximum-flow problem, and in turn, further reduce it to the minimum-cost perfect matching problem. But the algorithm is infeasible for practical genome sizes, and hence they propose a greedy heuristic to compute approximate solutions, that as shown in the results, achieves quite close results to the optimal algorithm.

Overall Assessment:

The proposed algorithm, and the introduction of the relaxation of the original SPSS problem, are interesting. But, the demonstrated output metrics—mainly the compression ratio (the Cumulative Length or CL size of the representation)—are not particularly convincing. The metrics do not show significant advantages of using the matchtigs representation over the simplitigs / UST-tigs / maximal path cover constructs existing in the literature. Besides, the resulting compression metrics are mostly marginal over these other representations. It can obviously be argued that the results are completely determined by the corresponding graph structures / topologies, since the minimum CL size is unique; and hence, the output metric has nothing to do with the algorithm but rather the graph structure. In that regard, the proposed method serves its purpose of computing the exact solution of their proposed problem. In this case, there must be an editorial determination of whether or not the results would appeal to the wide readership of this journal.

I have several concerns regarding the practical applications of matchtigs to the problems explored, and a few doubts about a subset of the experiments themselves.

Major Issues:

- While matchtigs achieve optimal results for the minimum k-mer set representation problem, giving up the unique existence of each k-mer in the output representation seems to pose non-trivial difficulties in downstream applications. Of course, matchtigs do not need to ensure such unique existences as per the modified problem definition. But, I cannot easily think of straightforward genomic applications apart from e.g. the "k-mer presence/absence" problem, where this representation would be plug-and-play suitable without non-trivial modifications in the corresponding tool / data structure.

- The practical benefits of the approach are not sufficiently clear. I would urge the authors to show some better use-case where their representation could have substantial improvements in the pipeline—this should be worthwhile to demonstrate the practical utility of the construct and the proposed tool.

- The k-mer based short read query experiment is explained clearly, but I have serious reservations about its underlying details. Given a set of input sequences, the Bifrost tool [2] constructs a compacted de Bruijn graph over the collection, and builds a minimizer-based index over the unitigs of the graph. This overview can be gleaned from both the associated paper and the corresponding GitHub repository. Thus, whether Bifrost is fed as input a collection of unitigs / simplitigs / UST-tigs / matchtigs, the index should be the same in absence of color information, since the underlying compacted de Bruijn graph is identical for all these different "tigs"-collections. Of course, the construction time may differ, as the different representations are of different input size. Yet, the resulting compacted de Bruijn graph index constructed by Bifrost should be the same. If the different sequences in each representation are said to have some unique color, then the Bifrost index need not be the same. The different representations would induce different colorings in the index, but such a coloring scheme over the 'tigs instead of the references does not have any immediately-apparent biological significance. Assuming that the authors did not use the colored index, it is not understandable to me how / why the indices are different for the different representations, and how the authors achieve their mentioned speedup, given that all the representations induce the same compacted de Bruijn graph for the Bifrost algorithm. If the colored variant of the de Bruijn graph is constructed, then the comparison is not "apples-to-apples" and the biologically interpretation of the colors is not clear. In general, the causal mechanism for the improved query speed should be better understood and explained, and, ideally, the scripts/code used to carry out the benchmark should be made available.

Minor issues:

- The abstract says that the authors propose a polynomial time algorithm for a problem "which was previously posed as a potentially NP-hard open problem". The actually posed problem is still open, and the authors solve a relaxed version of such. Thus, the claim should be adjusted accordingly; e.g. that a polynomial time algorithm is proposed for "a relaxation of a problem which was previously posed as a potentially NP-hard open problem."

- The last paragraph in the Background section uses "greedy matchtigs", without first introducing this concept.

- The String Count or SC metric is used a lot throughout the experiments and the discussions. Given that the proposed problem is to find the minimum Cumulative Length (CL) value, the significance / practicality of the SC metric should be discussed in the manuscript. In which contexts is this metric important or meaningful? Is it ever worthwhile to minimize SC at the expense of CL?

- The datasets used for the experiments seem a bit small. Some larger datasets, for example, a few mammalian / human genomes collection can be added to the experiments to show if the trends in the results continue scaling. Perhaps on larger or more repetitive genome collections, the practical gains from matchtigs will be larger.

- Figure 2 (illustrating the algorithm) is rather confusing.

- The accounting for self-complemental arcs / nodes in Section 5.5 is not easy to interpret, and I initially found it somewhat confusing to follow. The treatment could be made clearer (or, the discussion could be moved to the supplementary sections, with a brief summary put in the main text).


References

[1] Amatur Rahman and Paul Medvedev. Representation of k-mer sets using spectrum preserving string sets. In Russell Schwartz, editor, Research in Computational Molecular Biology, pages 152-168, Cham, 2020.
[2] Guillaume Holley and Páll Melsted. Bifrost: highly parallel construction and indexing of colored and compacted de bruijn graphs. Genome Biology, 21(1):249, September 2020.

**Reviewer 2**

The authors present a manuscript describing a polynomial time algorithm for the computation of an optimal minimum plain text representation of a k-mer set. Applying a variant of the Directed Chinese Postman Problem together with min-cost perfect matching, the approach computes a string representation of shortest length (not necessarily smallest number of strings). In addition to the optimal implementation, the authors also provide a greedy heuristic that is also practically feasible and shows close to optimal results in the presented experiments. The method is evaluated on a set of raw sequencing and assembled genome data from different organisms. The resulting matchtigs can be directly fed into downstream applications, translating the shorter representation length into speedup for query on the original input. The method can benefit all approaches that rely on a space-frugal representation of the k-mer spectrum of a given string set. All code and experimental data is provided.

The manuscript is very well written, both language- and structure-wise and I very much enjoyed reading it. The only recommendation I would make (if possible) is to either alter the general structure to put the description of methods first or to have a small intro paragraph introducing the notation / vocabulary used in the text. For instance, the concepts of biarcs and biwalks are used in the text before they are properly defined in the methods section.

Other than that, I only have a small selection of minor points:

* page 14, lines 18-19: Can it be excluded that the biwalk contains multiple subsequences of repeating nodes? I assume this is excluded by construction, but could be probably mentioned here.
* page 19, line 10: Typesetting issue of (b^-1m a^-1) - instead of (b^{-1}, a^{-1})
* additional_file_1, Figure 1: The black lines describing the #unitigs and avg. unitig length don't match the nature of the lines in the plots.
* additional_file_3, Figure 1: The genome length of Neisseria gonorrhoeae is missing the unit. Also, the same mismatch between legend and plots for black lines as in additional_file_1
* software: installation and setup worked flawlessly. Maybe some more functionality increasing convenience of use could be added, e.g. the ability to process gzip-compressed input files.

**Reviewer 3**

Matchtigs: Minimum plain text representation of kmer sets


This paper proposes a solution to provide the minimum cumulative length (CL) for representing unitigs allowing the repetition of kmers. This problem is np-hard in the presence of the constraint that allows representing each kmer exactly once, but the authors prove that allowing minimum number of repetitions of kmers, the problem is reduced to a polynomial classic problem and interstingly, the cumulative length could be even smaller. The final list of strings are called "minimum spectrum preserving string set (SPSS with kmer repetition)" which is the same name used previously to represent such a set with the modification of allowing repeated kmers in this paper. They reduce the problem to the bidirected partial-coverage Chinese postman problem which can eventually be solved using an integer linear program. The partiallity and bidirectionality of the graph changes the original min-cost flow problem.
However, with all the considerations to make the size of the final graph small, it is still not an efficient algorithm here (orders of quadratic number of arcs/links in the graph wrt the nodes). Therefore, they reduce the problem to a min-cost matching instance (similar to the original suggestion for solving Chinese postman problem) and solve it using Blossom V. Along this process of conversion of the graph, it is required to find min-cost paths between some specific nodes (which they call sources and sinks based on the definition). The authors solve this problem by applying Dijkstra in parallel for the many-to-many node shortest path finding.
Furthermore, they propose a greedy method as a replacement to the min-cost matching to keep a suboptimal set of shortest paths between source and sink nodes as part of the final SPSS. They show in their results that practically the suboptimality is negligible (and close to optimal result) considering the gain in reducing the computation time and memory.
The input to the algorithm is a list of Unitigs which are already built from converting a de Bruijn graph to a compacted de Bruijn graph. They use BCALM2 to build input Unitigs and then apply their algorithm on top of it.


The main idea of the paper is very interesting which is rather than forcing the kmers to be unique, we can benefit repeated kmers to gain a smaller final size of the set of kmer strings. I think this opens the door to a new set of algorithms (including downstream applications such as assembly, alignment and mapping) that use this idea, however currently as I know most of these kmer-based algorithms actually rely on the uniqueness of the kmers as a major factor in their design.
Interestingly, the improvements in SC measurement are more considerable compared to CL (which is the point of minimization in the algorithm) compared to previous work. I would say the improvement in CL is small considering the time and memory overhead.
It is nice that the authors get query improvement over Bifrost with only giving their output gfa as the input to Bifrost, but Bifrost query is already so inefficient compared to the state-of-the-art algorithms that requires orders of magnitude improvement rather than 1.3X.
Understandably, the issue of big CL for read files and limitation of the algorithm to work on large read collections is also still there as the unitigs are fragmented and small in reads. So the main improvement is over collections of similar genomes where Bifrost also has the best performance. The datasets selected for the result section also show this issue as also mentioned in the paper.
The pipeline is well-described although I would say there is more details required about previous work and algorithms used for a reader that is either not fully familiar with the field or even partially familiar with some of the work to easily follow the pipeline.
Using Rust as the implementation language is a bonus to the work.


Major:
The title conveys that you have reached the np-hard problem solution which is not the case. I'd suggest you change it from ".. kmer sets" to ".. kmer multisets" and correct "kmer set" to "kmer multiset" inside the text.
Also, I know that you clearly mention that you've adopted the SPSS from previous work and modified the concept a bit, but still I feel if you choose a specific name for list of string outputs it would be better specifically for future work.


This method seems not to be memory-efficient for reads due to the high number of unitigs (as mentioned in the text as well), but also the choice of the shortest-path algorithm, dijkstra. There are both algorithmic and engineering improvements to that. Dijsktra can be replaced with a low-memory algorithm or a variation of the algorithm specifically as the graph is degree-bound. Also since the direct distance values between nodes are limited (if I'm not wrong between 1 to k-1) you can put arcs into buckets (bucket sort) rather than using a distance array and this way you can load buckets one by one into memory which is way more efficient (refer to paper "An incrementally updatable and scalable system for large-scale sequence search using the Bentley-Saxe transformation"). Also you mention in the text that distance array is linear in number of threads. Does that mean that each thread is loading the distance array? You can change that to a shared resource with locks.


Although you have mentioned in different sections how you keep optimality of the CL (allowing kmer repetition), I would suggest you dedicate a specific section to that where you put all the proofs in one place as it is hard for the reader to track it. Proofs such as the minimality of the matching problem and the Chinese postman problem and minimum Eulerisation including the changes made to the balance constraint equation.


I was confused about making the graph complete and still do the Eulerisation. Isn't a complete graph eulerian already? I understand the necessity of finding a minimum partial eulerian cycle but not eulerising a complete graph. Is it the case that we will eventually have a complete graph with extra copies of some edges/arcs? Please explain in more details (better if with a figurative example) how these two are combined.


Figure 2 for me was very hard to follow. I definitely could not catch anything about the algorithm before reading the text and it was still hard to grasp the idea even after reading all the sections. Please try to make the figure more understandable either through a different example or changes in the figure. For example
you can make one of the nodes a, b, c, or d have a different imbalance value (other than |1| for all)
Rather than just mentioning the min-cost paths values, show them in the figure
Show the decision step where you break the breaking arc or dummy edges

Minor:
The figures are not designed for color-blind people. Use patterns rather than/as well as colors to show paths etc.
Section 2.2. The last sentence of third paragraph and first sentence of fourth paragraph repeat the same concept.
Section 2.2. Fourth paragraph: The fact that optimal solution for CL is producing a worse SC compared to the suboptimal one is actually very interesting and worth expanding, explaining, and investigating. Please add some description in that regards.
Is k = 51 a random choice or based on some heuristics? Please explain (maybe use additional-file 2 results for explanation)
Section 5.2. Last paragraph: Does that mean that BCALM2 is part of your pipeline implementation or one needs to run it first? I noticed that you support both fasta and gfa as input to your pipeline so I guess it's the former, but please specify.
Section 5.4. Second paragraph, mention the graph is "complete", adding the rest of the remaining edges as breaking arcs.
Section 5.4. Second paragraph, bring a short explanation on why you choose cost of "k-1" for breaking arcs the first time you mention it (here).
Section 5.4. Add a figure to explain the last paragraph and show why and in which cases you need those two types of cutting the walks.
Section 5.5. Second paragraph, make "variable" singular: variable x_e is interpreted … and variable c_e denotes ..
Section 5.5. Third paragraph: enumerating the three changes in bullet points could be more eye-catching and easier to follow
Section 5.5. Third paragraph: i.e. that the flow of each arc → i.e. the flow of each arc (remove "that")
Section 5.5. Last paragraph: rewrite the part where you explain why most of the source-sink arcs are not needed: "otherwise, the longer source-sinc path .. a solution with lower costs." It is complicated and has small grammatical errors.
Section 5.6. First paragraph: we can further restrict it → not clear what "it" is referring to (probably the algorithm?)

**Authors’ response**

We thank the reviewers for their comments which improved the presentation of the paper, the implementation and the experimental results. We addressed all comments, as follows.

\## Our general comments about this revision

- To be able to answer all the reviewer's comments, we have invited a new coauthor, Giulio Ermanno Pibiri. He is the author of SSHash, which is a state-of-the-art kmer dictionary, whose preprint was published only after our submission to Genome Biology. We modified SSHash such that its "Lookup" query is now able to handle duplicate kmers, and measured query speedups of up to 2.10 over previous work. Since the reviewers were not satisfied with using Bifrost as an application, we have moved the Bifrost experiment into an additional file.

- During the review, a paper was published that proves that the sister problem without repeating kmers is actually linearly solvable. We have updated the paper with the respective references and literature review.

- We have optimised the memory consumption of our tool, nearly halving the memory used to compute greedy matchtigs on the most resource-intensive dataset (B. mori reads).

- We had wrongly used the term "simplitigs" for the strings produced by ProphAsm. The paper introducing simplitigs actually defines simplitigs as a plain text representation without kmer repetitions, but does not tie the term to any specific algorithm to optimality. We have changed our paper to respect the original definition.

Reviewer #1

A. , the demonstrated output metrics---mainly the compression ratio (the Cumulative Length or CL size of the representation)---are not particularly convincing. The metrics do not show significant advantages of using the matchtigs representation over the simplitigs / UST-tigs / maximal path cover constructs existing in the literature. Besides, the resulting compression metrics are mostly marginal over these other representations. It can obviously be argued that the results are completely determined by the corresponding graph structures / topologies, since the minimum CL size is unique; and hence, the output metric has nothing to do with the algorithm but rather the graph structure. In that regard, the proposed method serves its purpose of computing the exact solution of their proposed problem. In this case, there must be an editorial determination of whether or not the results would appeal to the wide readership of this journal.

**Answer:** We understand that the improvement in CL ratio does not look that big in itself. However we would like to stress that downstream applications, like kmer streaming queries, also benefit a lot from a lower string count, as it reduces the fraction of index lookups as now described in Section 2.5. As seen in that section, we have complemented Bifrost with a state-of-the-art kmer query tool, SSHash. Its query speedup (2.10) is now significantly larger than the reduction in CL (1.19) for the Salmonella pangenome, and significantly larger than the query speedup (1.29) of Bifrost on the smaller E. coli pangenome.

Further, our technique falls into the category of "preprocessing algorithms" which are typically ran only once and then their output can be used to speedup arbitrarily many executions of arbitrarily many different downstream applications.

Also, consider that any non-plain-text representation would require decompression on each access, while in a plain-text representation each kmer is simply a slice of memory (potentially of bits instead of bytes, if bases are encoded with 2 bits instead of ASCII). Our representation is the smallest that does not require decompression, which puts it to a special point in the decompression-time to storage-space tradeoff. Any smaller means an increase in access times, while access times will stay the same for a larger representation.

Lastly, to (indirectly) underline the interest to the problem of optimal plain-text representations of kmer sets, note that while this paper was under review, two authors of this paper (Sebastian Schmidt and Jarno Alanko) proved that a minimum representation without repeated kmers is achievable in linear time. This was published in the proceedings of WABI 2022 (Workshop for Algorithms in Bioinformatics) which won the WABI 2022 Best Paper award (name: "Eulertigs: minimum plain text representation of k-mer sets without repetitions in linear time"). We believe that the current submission analogously closes the problem when allowing repetitions, both with an optimal algorithm, and with an efficient heuristic, that is very close to optimum.

B. While matchtigs achieve optimal results for the minimum k-mer set representation problem, giving up the unique existence of each k-mer in the output representation seems to pose non-trivial difficulties in downstream applications. Of course, matchtigs do not need to ensure such unique existences as per the modified problem definition. But, I cannot easily think of straightforward genomic applications apart from e.g. the "k-mer presence/absence" problem, where this representation would be plug-and-play suitable without non-trivial modifications in the corresponding tool / data structure.

**Answer:** First, note that Bifrost can use matchtigs without any modifications to itself. Bifrost goes beyond just simple $k$-mer presence/absence by supporting pseudoalignment queries. Further, to address the problem of duplicate $k$-mers in downstream applications, we have modified our tool to also output a bitvector marking all duplicated kmers (such that exactly one copy of each kmer is unmarked). This may help downstream applications to integrate a representation with repeated kmers. Finally, we have now also modified a subset of the tool SSHash, which does not support repeated kmers by default. The modified variant SSHash-Lite allows to use repeated kmers, and the modification was very simple (described in Section 2.5).

Modifying the complete SSHash (which maps kmers to a range of consecutive indices) would not be more difficult. By default, SSHash would map duplicate kmers to separate indices. But given the duplication bitvector, the duplicate indices can be filtered and the "holes" in the consecutive range can be closed with a standard rank-query on the bitvector.

C. The practical benefits of the approach are not sufficiently clear. I would urge the authors to show some better use-case where their representation could have substantial improvements in the pipeline---this should be worthwhile to demonstrate the practical utility of the construct and the proposed tool.

**Answer:** We have extended our experiments with kmer queries to be more convincing. We have added larger datasets, that in turn yield better speedups over previous work (2.10, as opposed to 1.29 that we got before). While not all datasets are significantly compressible using matchtigs, our results here show that especially for datasets with a very tangled de Bruijn graph, good speedups are possible. The demonstrated speedups carry over to any application using kmer queries at their core, and they also motivate that other applications may benefit by the same principle -- less size (CL) means less memory, and less strings (SC) mean less overhead for processing.

D. The k-mer based short read query experiment is explained clearly, but I have serious reservations about its underlying details. Given a set of input sequences, the Bifrost tool [2] constructs a compacted de Bruijn graph over the collection, and builds a minimizer-based index over the unitigs of the graph. This overview can be gleaned from both the associated paper and the corresponding GitHub repository. Thus, whether Bifrost is fed as input a collection of unitigs / simplitigs / UST-tigs / matchtigs, the index should be the same in absence of color information, since the underlying compacted de Bruijn graph is identical for all these different "tigs"-collections. Of course, the construction time may differ, as the different representations are of different input size. Yet, the resulting compacted de Bruijn graph index constructed by Bifrost should be the same. If the different sequences in each representation are said to have some unique color, then the Bifrost index need not be the same. The different representations would induce different colorings in the index, but such a coloring scheme over the 'tigs instead of the references does not have any immediately-apparent biological significance. Assuming that the authors did not use the colored index, it is not understandable to me how / why the indices are different for the different representations, and how the authors achieve their mentioned speedup, given that all the representations induce the same compacted de Bruijn graph for the Bifrost algorithm. If the colored variant of the de Bruijn graph is constructed, then the comparison is not "apples-to-apples" and the biologically interpretation of the colors is not clear. In general, the causal mechanism for the improved query speed should be better understood and explained, and, ideally, the scripts/code used to carry out the benchmark should be made available.

**Answer:** It seems like our explanation of how we use Bifrost was not clear enough here. The Bifrost binary actually has three subcommands, of which we use only one. Bifrost can build a compacted DBG from a set of input strings, it can update a compacted DBG by adding new input strings, and it can query a given compacted DBG. We only use the *query* part where it queries a given compacted DBG. When used in the intended way, the query part takes a set of query sequences as input, as well as a compacted DBG in GFA format. It then first builds the query index and then uses the index to execute the queries (since it rebuilds the index every time, we measure the actual query time separately). We "misuse" Bifrost by not passing it a compacted DBG, but other sorts of tigs, i.e. matchtigs, greedy matchtigs, simplitigs and UST-tigs. Bifrost does not build a compacted DBG from this input, but only builds the query index and executes the query. The query index is a minimizer hash table, and does not make use of any topological information of the DBG, this is why the query then still works correctly. The query index is actually different when building it over different tigs, and this allows Bifrost to perform differently when passed different tigs for building the query index.

We have now moved our experiment with Bifrost into Additional file 6 and expanded our explanation of the query experiment there. Further, our new Section 2.5 contains a detailed explanation of how matchtigs speed up SSHash-Lite as well.

E. The abstract says that the authors propose a polynomial time algorithm for a problem "which was previously posed as a potentially NP-hard open problem". The actually posed problem is still open, and the authors solve a relaxed version of such. Thus, the claim should be adjusted accordingly; e.g. that a polynomial time algorithm is proposed for "a relaxation of a problem which was previously posed as a potentially NP-hard open problem."

**Answer:** Since the Editor indicated that the abstract must fit 100 words, we found it best to simply remove this part from the abstract, now shortened to 100 words.

F. The last paragraph in the Background section uses "greedy matchtigs", without first introducing this concept.

**Answer:** We removed the term and instead refer to our greedy heuristic that we mentioned about in the beginning of the "Our contributions" paragraph.

G. The String Count or SC metric is used a lot throughout the experiments and the discussions. Given that the proposed problem is to find the minimum Cumulative Length (CL) value, the significance / practicality of the SC metric should be discussed in the manuscript. In which contexts is this metric important or meaningful? Is it ever worthwhile to minimize SC at the expense of CL?

**Answer:** Added a few sentences after the metrics are introduced about the significance of SC and the tradeoff between the two.

H. The datasets used for the experiments seem a bit small. Some larger datasets, for example, a few mammalian / human genomes collection can be added to the experiments to show if the trends in the results continue scaling. Perhaps on larger or more repetitive genome collections, the practical gains from matchtigs will be larger.

**Answer:** We added a pangenome of ~309k references of Salmonella, as well as a pangenome of 2505 human references. While the human pangenome is not as tangled, the Salmonella pangenome appears to be, and hence achieves good CL and SC reductions. Still, it is less tangled than the short read datasets, which contain a lot of erroneous kmers. However, it is visible that the improvements for kmer-based queries are larger for the Salmonella pangenome, motivating that on larger datasets the improvements in downstream applications continue to scale.

I. Figure 2 (illustrating the algorithm) is rather confusing.

**Answer:** Please see our answer to 3.M, where reviewer 3 raised the same issue.

J. The accounting for self-complemental arcs / nodes in Section 5.5 is not easy to interpret, and I initially found it somewhat confusing to follow. The treatment could be made clearer (or, the discussion could be moved to the supplementary sections, with a brief summary put in the main text).

**Answer:** We have chosen to keep this in the main text. We have extended the explanation a little, as well as corrected errors in the formulation described in Section General (which may have caused confusion). We also added a figure explaining the case with the self-complemental biarc.

Reviewer #2

A. The only recommendation I would make (if possible) is to either alter the general structure to put the description of methods first or to have a small intro paragraph introducing the notation / vocabulary used in the text. For instance, the concepts of biarcs and biwalks are used in the text before they are properly defined in the methods section.

**Answer:** We added a short subsection at the beginning of the results section explaining our graph model.

B. page 14, lines 18-19: Can it be excluded that the biwalk contains multiple subsequences of repeating nodes? I assume this is excluded by construction, but could be probably mentioned here.

**Answer:** As mentioned in the general comments, this part was actually wrong. We have fixed it, and now break the biwalk only at breaking biarcs

.

C. page 19, line 10: Typesetting issue of (b^-1m a^-1) - instead of (b^{-1}, a^{-1})

**Answer:** Fixed.

D. additional_file_1, Figure 1: The black lines describing the ##unitigs and avg. unitig length don't match the nature of the lines in the plots.

**Answer:** Fixed.

E. additional_file_3, Figure 1: The genome length of Neisseria gonorrhoeae is missing the unit. Also, the same mismatch between legend and plots for black lines as in additional_file_1

**Answer:** Added the missing unit and fixed the legend and plot lines.

F. software: installation and setup worked flawlessly. Maybe some more functionality increasing convenience of use could be added, e.g. the ability to process gzip-compressed input files.

**Answer:** Added the ability to process gzip-compressed input files as well as producing gzip-compressed output.

Reviewer #3

A. This problem is np-hard in the presence of the constraint that allows representing each kmer exactly once, but the authors prove that allowing minimum number of repetitions of kmers, the problem is reduced to a polynomial classic problem and interstingly, the cumulative length could be even smaller.

**Answer:** This problem has actually turned out be linearly solvable, see the updated related work section for details.

B. The main idea of the paper is very interesting which is rather than forcing the kmers to be unique, we can benefit repeated kmers to gain a smaller final size of the set of kmer strings. I think this opens the door to a new set of algorithms (including downstream applications such as assembly, alignment and mapping) that use this idea, however currently as I know most of these kmer-based algorithms actually rely on the uniqueness of the kmers as a major factor in their design.

**Answer:** Please see our answer to 1.B, where reviewer 1 raised the same issue.

C. I would say the improvement in CL is small considering the time and memory overhead.

**Answer:** Please see our answer to 1.A, where reviewer 1 raised the same issue.

D. It is nice that the authors get query improvement over Bifrost with only giving their output gfa as the input to Bifrost, but Bifrost query is already so inefficient compared to the state-of-the-art algorithms that requires orders of magnitude improvement rather than 1.3X.

**Answer:** Bifrost was published one year before our submission to this journal, hence we would consider it state-of-the-art for the purpose of this submission. Consider also, that Bifrost simultaneously performs queries and writes query results to a file, hence Bifrost queries may look slower, even though the query algorithm itself is fast.

Nevertheless, we have now also included query results with a modified version of SSHash, whose preprint was first published one month after our submission to this journal.

E. Understandably, the issue of big CL for read files and limitation of the algorithm to work on large read collections is also still there as the unitigs are fragmented and small in reads. So the main improvement is over collections of similar genomes where Bifrost also has the best performance. The datasets selected for the result section also show this issue as also mentioned in the paper.

**Answer:** We have improved the memory requirements of our algorithm, and managed to reduce the memory required for computing greedy matchtigs of the most extreme dataset (B. mori short reads with a minimum abundance of 1) from 227 to 123 GiB. For a smaller dataset, C. elegans, the memory required to compute greedytigs dropped from 73.3 to 41.0 GiB. This was possible without slowing down computation time (even without the optimisation mentioned next). We additionally optimised the loading and building of the arc-centric de Bruijn graph, utilising the extra information about unitig links that is present in BCALM2's fasta output.

F. The pipeline is well-described although I would say there is more details required about previous work and algorithms used for a reader that is either not fully familiar with the field or even partially familiar with some of the work to easily follow the pipeline

**Answer:** We added more details about the previous algorithms to the related work paragraph. Also, we added a figure about our pipeline in the end in the *Experimental evaluation* subsection, with some explanation.

G. The title conveys that you have reached the np-hard problem solution which is not the case. I'd suggest you change it from ".. kmer sets" to ".. kmer multisets" and correct "kmer set" to "kmer multiset" inside the text.

**Answer:** We disagree with this proposal. First, we do not agree that the title conveys that kmers would be unique in the representation. That is, while we do represent kmer sets, we do not represent them by storing only one copy of each kmer, but at least one. Semantically, what we store is still a set, as there is no further meaning to a kmer occurring more than once in the representation. Kmers are either not part of the set if they dont appear in plain text in the representation, or they are part of the set if they appear at least once. Changing the name to multisets would convey that there would be a meaning to kmers appearing more than once, which is false.

H. Also, I know that you clearly mention that you've adopted the SPSS from previous work and modified the concept a bit, but still I feel if you choose a specific name for list of string outputs it would be better specifically for future work.

**Answer:** We have now adopted the term simplitigs to describe simplitigs as well as the SPSS as defined by Rahman and Medvedev's paper. But we decided to stick with redefining the term SPSS, and added more explanation in the paper. In a nutshell, the SPSS by Rahman and Medvedev should have been called a multispectrum preserving string set, as they define the spectrum as a multiset. Usually the spectrum is defined as a set though. If you do not agree with this, we can still come up with a different term.

I. This method seems not to be memory-efficient for reads due to the high number of unitigs (as mentioned in the text as well), but also the choice of the shortest-path algorithm, Dijkstra. There are both algorithmic and engineering improvements to that. Dijsktra can be replaced with a low-memory algorithm or a variation of the algorithm specifically as the graph is degree-bound. Also since the direct distance values between nodes are limited (if I'm not wrong between 1 to k-1) you can put arcs into buckets (bucket sort) rather than using a distance array and this way you can load buckets one by one into memory which is way more efficient (refer to paper "An incrementally updatable and scalable system for large-scale sequence search using the Bentley-Saxe transformation").

**Answer:** Our many-to-many Dikstra works by executing a set of one-to-many Dijkstras in parallel. Each has their own priority queue and distance array, and these cannot be shared, as depending on the source, a node has different entries in the distance array and priority queue. Also, we execute a lot of one-to-many Dijkstra queries, which are so fast each that using external memory is unlikely to yield a speedup, especially if that external memory is used by 28 or more threads in parallel. However, to decrease memory, we have now replaced the distance array in Dijkstra with hash map (mapping from node_index to distance from source), which decreases memory consumption, but surprisingly does not increase runtime (while the runtimes did increase for most experiments from the last iteration to the current iteration of the paper, this is because we have switched to $k=31$ for these datasets for easier comparison of the results on the different datasets).

Since the runtime did not decrease when exchanging the distance array, we have decided against exchanging the priority queue (which could use a bucketing strategy similar to what you described, as opposed to the heap it is using now). Likely, the algorithm is currently bottlenecked by the random memory accessed caused by traversing the graph.

J. Also you mention in the text that distance array is linear in number of threads. Does that mean that each thread is loading the distance array? You can change that to a shared resource with locks.

**Answer:** Each thread has its own version of the distance array, as the entry of a node in the distance array depends on the source of the search. The entry of a node in the distance array stores the length of the shortest currently known path from the source to the node. So since each thread computes Dijkstras with pairwise distinct sources, no two threads can share a distance array, as they might have a different value for the same node. Hence, we need a separate distance array (or equivalent data structure) for each thread.

K. Although you have mentioned in different sections how you keep optimality of the CL (allowing kmer repetition), I would suggest you dedicate a specific section to that where you put all the proofs in one place as it is hard for the reader to track it. Proofs such as the minimality of the matching problem and the Chinese postman problem and minimum Eulerisation including the changes made to the balance constraint equation.

**Answer:** We added Additional file 8, containing a complete proof of optimality of our algorithm. While writing the proof, we fixed the following minor mistakes.

We found one edge case where the algorithms output was not optimal when the graph can be Eulerised without using any breaking arcs, but with using some joining arcs. Specifically, by allowing the rural postman walk to contain zero breaking arcs, it was possible that a graph admits different optimal rural postman walks of which only some produce optimal solutions to our problem. For example, there could be one optimal rural postman walk with two subwalks of repeated arcs of length 2, and one where it contains one subwalk of repeated arcs of length 1, and one of length 3. When breaking the walks by removing maximal subwalks made of repeated arcs, this would result in the second walk being one character shorter than the first, because in the second walk three arcs can be removed, but in the first just two can be removed. Our algorithm would have just returned an arbitrary of the two walks, therefore it was not optimal.

We have fixed this now by requiring the rural postman walk to cover at least one breaking arc, and we have propagated this constraint through the flow formulation to the matching instance. This has the additional advantage, that the costs optimised when finding the walk are exactly the same as the CL of the final output of the algorithm. There was a mistake in the manuscript in the flow formulation where the bi-imbalance introduced by self-complemental arcs was only computed correctly for the original graph, but not for the flow in the graph, which has now been fixed. There was a mistake in the manuscript in the flow formulation where the costs of self-complemental biarcs were counted only once, but the costs of other biarcs were counted twice. This has been fixed by using canonical kmers, such that we now count the costs of other biarcs only once as well.

We have so far only presented our algorithm for a graph which is connected. If a graph has multiple connected components, the algorithm does not work. In practice, we solved that by handling the components separately, which we now also described in the paper, together with an argument for why that does not influence optimality.

We have fixed these errors in the manuscript and in the implementation.

L. I was confused about making the graph complete and still do the Eulerisation. Isn't a complete graph eulerian already? I understand the necessity of finding a minimum partial eulerian cycle but not eulerising a complete graph. Is it the case that we will eventually have a complete graph with extra copies of some edges/arcs? Please explain in more details (better if with a figurative example) how these two are combined.

**Answer:** There was a mistake in the description, falsely stating that the graph would be made complete. What really happens is that the breaking arcs are added between all pairs of nodes, and the resulting graph is a multigraph. But the breaking arcs are actually optional and not all of them are supposed to be part of the solution to our variant of the Chinese postman problem, but only those that make the solution minimum.

We have corrected the description and added more detail by formally defining the "transformed" graph.

M. Figure 2 for me was very hard to follow. I definitely could not catch anything about the algorithm before reading the text and it was still hard to grasp the idea even after reading all the sections. Please try to make the figure more understandable either through a different example or changes in the figure. For example you can make one of the nodes a, b, c, or d have a different imbalance value (other than $|1|$ for all). Rather than just mentioning the min-cost paths values, show them in the figure. Show the decision step where you break the breaking arc or dummy edges.

**Answer:** We have reworked the figure. The new example now has less nodes overall and a node with imbalance 2. We have spelled out the min-cost paths, and in the caption stated the formula for computing the costs of each path, as well as explained where the costs come from. We have made extra boxes showing the biEulerian circuit as well as the broken circuit. To save space we have decided to not go into any details about the matching problem, but instead just explained what it does on a higher level.

N. The figures are not designed for color-blind people. Use patterns rather than/as well as colors to show paths etc.

**Answer:** Since Figure 1 would require 7 different sets of dash patterns, we have decided to instead make the thick lines representing the tigs non-overlapping, such that even without color information they should be tracable by following the shape. When reworking Figure 2, we ensured that all lines that are distinguished by colors are also distinguished by dash patterns. We made the thick lines in Figure 3 distinguishable by dash patterns. We made all the figures in the additional files color blind friendly by using dash patterns and fill patterns on top of colors.

O. Section 2.2. The last sentence of third paragraph and first sentence of fourth paragraph repeat the same concept.

**Answer:** We removed the duplicate.

P. Section 2.2. Fourth paragraph: The fact that optimal solution for CL is producing a worse SC compared to the suboptimal one is actually very interesting and worth expanding, explaining, and investigating. Please add some description in that regards.

**Answer:** We added explanation into this paragraph, and additionally in the ends of the corresponding sections in the methods "Solving the min-cost integer flow formulation with min-cost matching" and "Efficient computation of the greedy heuristic".

Q. Is k = 51 a random choice or based on some heuristics? Please explain (maybe use additional-file 2 results for explanation)

**Answer:** The choice of $k$ around 51 for human-sized genomes is quite common, as e.g. in "De novo assembly and genotyping of variants using colored de Bruijn graphs". However, what is a "good" $k$ seems to depend on the specific analysis, as e.g. in "Toward fast and accurate SNP genotyping from whole genome sequencing data for bedside diagnostics", $k = 32$ is used to reduce the number of errors in kmers. We have now changed to use the same $k = 31$ for all genomes in the main matter, to make the results more comparable and easier to interpret. In the additional files we continue to give a range of $k$ values.

R. Section 5.2. Last paragraph: Does that mean that BCALM2 is part of your pipeline implementation or one needs to run it first? I noticed that you support both fasta and gfa as input to your pipeline so I guess it's the former, but please specify.

**Answer:** We expect a de Bruijn graph as input, we have now clarified that our tool does not build the de Bruijn graph itself.

S. Section 5.4. Second paragraph, mention the graph is "complete", adding the rest of the remaining edges as breaking arcs.

**Answer:** See the answer to 3.L.

T. Section 5.4. Second paragraph, bring a short explanation on why you choose cost of "k-1" for breaking arcs the first time you mention it (here).

**Answer:** Added a sentence for explanation

.

U. Section 5.4. Add a figure to explain the last paragraph and show why and in which cases you need those two types of cutting the walks.

**Answer:** We have updated and corrected the description of how the final circular walk is cut. Since the correct variant is much simpler (simply cut at all breaking arcs, as the new formulation guarantees that there is always at least one), we hope that it is understandable without figure.

V. Section 5.5. Second paragraph, make "variable" singular: variable x_e is interpreted ... and variable c_e denotes ..

**Answer:** Done.

W. Section 5.5. Third paragraph: enumerating the three changes in bullet points could be more eye-catching and easier to follow

**Answer:** We have enumerated the changes with bullet points.

X. Section 5.5. Third paragraph: i.e. that the flow of each arc → i.e. the flow of each arc (remove "that")

**Answer:** Done.

Y. Section 5.5. Last paragraph: rewrite the part where you explain why most of the source-sink arcs are not needed: "otherwise, the longer source-sinc path .. a solution with lower costs." It is complicated and has small grammatical errors.

**Answer:** We added a longer and more detailed description.

Z. Section 5.6. First paragraph: we can further restrict it → not clear what "it" is referring to (probably the algorithm?)

**Answer:** What was meant there was that flow moves from a source to a sink only via paths of length at most k-2 (or k-1 as elaborated afterwards). So we changed the "it" to "flow" to clarify.

**2^nd^ round**

**Reviewer 1**

The authors have addressed our previous concerns. We have a few small comments and queries:

* The k can be italicized in all the occurrences of k-mers in the text.

* In the experiments described in Sec. 2.3 (Compression of model organisms), when working with sequencing data, k-mer filtering has not been applied for some read sets; while it is done for the human reads with an abundance threshold of 10. What is the rationale of not using the filtering on some datasets, as opposed to the standard practice?

* At the last paragraph in page 3, the authors briefly discuss the impact of UST-tigs (from Rahman and Medvedev) and simplitigs (from Břinda et al) on CL and SC, opposed to unitigs. It might be worthwhile to note that Khan et al. 2022 also provide some comparative statistics on maximal path cover vs unitigs decomposition in their supplementary table 6.

* In table 3, the number of unique k-mers is reported for all the datasets except for the 2505x human refs, which should be present for completeness. Also, the RAM-usage on this dataset is reported as > 411GB, which seems quite high unless the unique k-mer count is really huge.

**Reviewer 2**

Thanks to the authors for providing answers to many of my concerns and correcting the manuscript accordingly. Happy to hear that some of the concerns led the authors to some corrections in the algorithm and code. The figures have also improved much better.

I am also happy to see the implementation and results for supporting kmer-based downstream applications such as kmer search which requires uniqueness of kmers.

However, looking at the results in general, I am still not convinced that the improvements are considerable. I understand that based on the proofs, we might be at the borders of improving performance (no further improvement), however, still the memory cost is considerable for marginally reducing the CL.

Major Issues:
I still insist that the title must be multi-set rather than set and am not convinced by the authors' response. The definition of multi-set does not come from the utility of the elements or associated properties but the sole repetition of them.

Also, I still believe no matter which definition is more accurate for SPSS, since it has been introduced a different way before, authors must consider choosing a new name for the type of strings they provide.


Minor corrections:
Figure 2 caption: The costs of each arc are → The cost of each arc is
Figure 2 caption: the costs of an arc are → The cost of an arc is
5.5. Last paragraph: incoming args with demands → incoming args which demands

**Authors’ response**

Reviewer #1

1. The k can be italicized in all the occurrences of k-mers in the text.

**Answer:** We have switched from kmer to $k$-mer, which is the more proper mathematical way of referring to substrings of length $k$.

2. In the experiments described in Sec. 2.3 (Compression of model organisms), when working with sequencing data, k-mer filtering has not been applied for some read sets; while it is done for the human reads with an abundance threshold of 10. What is the rationale of not using the filtering on some datasets, as opposed to the standard practice?

**Answer:** We have chosen unfiltered read datasets because we want our experiments to be independent of any possible preprocessing of the datasets. It would for example be unclear what a good value for minimum abundance for any of the datasets is, given that a good value may vary based on application. We have investigated the effect of varying minimum abundance in Additional file 1: Supplemental figure S2. The reason why we chose a minimum abundance of 10 for human is the extreme size of the dataset. Since the human genome is roughly an order of magnitude longer than the other two and the dataset has around 5x the coverage, its raw unique k-mer count is 114 billion, where the two other have only 1.35 billion and 3.66 billion. While it is possible to build unitigs using the state-of-the-art tool Cuttlefish 2, both ProphAsm (executed on the unitigs) and greedy matchtigs run out of memory even with 2TiB of RAM. When filtering the human read dataset with a min abundance of 10, then it has 2.78 billion unique k-mers, which can be handled by both tools. We have now mentioned this limitation in the paper.

3. At the last paragraph in page 3, the authors briefly discuss the impact of UST-tigs (from Rahman and Medvedev) and simplitigs (from Břinda et al) on CL and SC, opposed to unitigs. It might be worthwhile to note that Khan et al. 2022 also provide some comparative statistics on maximal path cover vs unitigs decomposition in their supplementary table 6.

**Answer:** Thank you for pointing that out. We have added a sentence to the last paragraph on page 3 mentioning Khan et al. 2022.

4. In table 3, the number of unique k-mers is reported for all the datasets except for the 2505x human refs, which should be present for completeness. Also, the RAM-usage on this dataset is reported as > 411GB, which seems quite high unless the unique k-mer count is really huge.

**Answer:** We have added the missing $k$-mer count. The high memory consumption stems from the fact that we use the `--unrestricted-memory` flag to speed up our experiments. Even with using that flag, Cuttlefish 2 takes around a day for each run. We have pointed that out now in the table caption.

Reviewer #3

1. Looking at the results in general, I am still not convinced that the improvements are considerable. I understand that based on the proofs, we might be at the borders of improving performance (no further improvement), however, still the memory cost is considerable for marginally reducing the CL.

**Answer:** It is true that the computation of greedy matchtigs comes at a high cost in terms of memory. However, matchtigs only need to be computed once per dataset, and then matchtigs can be used in arbitrary many analyses of that dataset, roughly halving the runtime of each analysis. Under that point of view, the memory cost is negligible, since the computed matchtigs can be used infinitely often.

2. I still insist that the title must be multi-set rather than set and am not convinced by the authors' response. The definition of multi-set does not come from the utility of the elements or associated properties but the sole repetition of them. Also, I still believe no matter which definition is more accurate for SPSS, since it has been introduced a different way before, authors must consider choosing a new name for the type of strings they provide.

**Answer:** We agree that in general one should avoid redefining terminology from other papers. However, to us, a "spectrum preserving string set" sounds more like a set of strings that has the same spectrum as some other set of strings. The name does not communicate any restriction on the set of strings, like that it must contain each $k$-mer exactly once.

We do have considered choosing a different name for the unrestricted concept anyways, but found it to be strange to have a longer name like "spectrum preserving string set with repetitions" for the more general concept with less restrictions.

We are strongly against naming our concept "spectrum preserving string multi-set", since mathematically, that would be a string multi-set with the attribute "spectrum preserving". But what we compute is explicitly not a string multi-set, since reporting any string twice would never be useful in our case.

We have explicitly mentioned that our SPSS is different from that defined by Rahman and Medvedev. To avoid confusion, we have stated this both in "Motivation" and "Related work" in the introduction, as well as in the methods where we formally define an SPSS.

3. Figure 2 caption: The costs of each arc are → The cost of each arc is

Figure 2 caption: the costs of an arc are → The cost of an arc is

**Answer:** When used as a noun, "cost" is a special case that refers to a single item in its plural form, but in its singular form it refers to cost that is composed of multiple costs (<https://kris-spisak.com/writing-tip-cost-vs-costs/>). In the cases mentioned, the costs of an arc can be viewed as the sum of the characters, or the sum of the costs of the characters, allowing for both forms to work.

4. 5.5. Last paragraph: incoming args with demands → incoming args which demands

**Answer:** The word "demands" here is a term from flow theory. The demand of a source or sink is the amount of flow that it "creates" or "destroys". We added a comma to make the sentence structure more clear
